# From bedside to bug side: clinical, haematological and genetic markers of antibiotic-resistant bacterial isolates from children admitted with sepsis in Kaduna State, Nigeria – a protocol for a cross-sectional study

**DOI:** 10.1136/bmjopen-2025-106612

**Published:** 2025-09-26

**Authors:** Sani Musa, Shamsudin Aliyu, Nuruddeen Bida Abdullahi, Hajara Lawal Khalid, Sadiq Kwaifa Salihu, Aliyu Umar Dahiru, Amal Aminu Muhammad, Kamilah Abdullahi, Suleiman Salisu, Summaiyah Ahmad Gumbi, Zainab Lamido Tanko, Hauwau Makarfi Umaru, Halima Bello-Manga, Livingstone Gayus Dogara, Abdullahi Musa, Ibrahim Kusfa Usman, Umar Waisu Lawal, David W Cleary, Jessica M A Blair

**Affiliations:** 1 Department of Microbes, Infections, and Microbiomes, College of Medicine and Health & Institute of Microbiology and Infection, University of Birmingham, Birmingham, UK; 2 Paediatrics and Child Health, Ahmadu Bello University & Teaching Hospital, Zaria, Kaduna, Nigeria; 3 Medical Microbiology, Ahmadu Bello University & Teaching Hospital, Zaria, Kaduna, Nigeria; 4 Paediatrics and Child Health, Ahmadu Bello University Teaching Hospital, Zaria, Kaduna, Nigeria; 5 Haematology and Blood Transfusion, Ahmadu Bello University Teaching Hospital, Zaria, Kaduna, Nigeria; 6 Paediatrics and Child Health, Barau Dikko Teaching Hospital, Kaduna, Nigeria; 7 Medical Microbiology, Barau Dikko Teaching Hospital & Kaduna State University, Kaduna, Nigeria; 8 Haematology and blood transfusion, Barau Dikko Teaching Hospital & Kaduna State University, Kaduna, Nigeria; 9 Haematology and Blood Transfusion, Ahmadu Bello University & Teaching Hospital, Zaria, Kaduna, Nigeria

**Keywords:** Child, Sepsis, Antibiotics, HAEMATOLOGY, Molecular diagnostics, MICROBIOLOGY

## Abstract

**Abstract:**

**Introduction:**

Sepsis and antibiotic resistance constitute a deadly synergy, causing the loss of millions of lives across the world, with their economic and developmental consequences posing a threat to global prosperity. Their impact is disproportionately felt in resource-limited settings and among vulnerable populations, especially children. A key challenge is prompt diagnosis and timely commencement of appropriate antibiotic therapies. These challenges are compounded in low-income and middle-income countries by a lack of comprehensive epidemiological data, with Nigeria being one such country for which it is lacking. Kaduna is the third largest state in Nigeria, with over 10 million inhabitants, of whom more than half are children under 14 years old. While bacterial sepsis and antimicrobial resistance (AMR) are recognised as a growing problem in the state, there are huge gaps in the current understanding of their aetiology. This project employs a cross-sectional design to investigate the clinical and haematological markers of paediatric sepsis, alongside determining the bacterial cause and prevalence of AMR at four high-turnover hospitals in Kaduna State, Nigeria. Further, whole-genome sequencing of isolated bacterial pathogens will be performed to determine the genetic features of resistance. This project represents the largest surveillance study of paediatric sepsis in Kaduna to date. Additionally, we aim to use the clinical, haematological, microbiological and genomic data to derive predictive models for sepsis causes, treatment strategies and patient outcomes.

**Methods and analysis:**

This is a hospital-based, cross-sectional study that will recruit up to 461 children with bacterial sepsis who were admitted at the two teaching and two general hospitals in Kaduna State, Nigeria. Children presenting with features of fever, subnormal temperature and body weakness would be recruited into the study and have their blood samples collected. The blood samples will be used for culture, complete blood count, HIV and malaria testing. Accordingly, we will capture clinical presentation, haematological characteristics, causative pathogen from blood culture and patient outcomes. Nutritional status, known congenital immunosuppressive diseases, HIV infection and malaria infection will also be determined and documented. The bacterial isolates will be phenotypically characterised for AMR and genotypically following whole genome sequencing. Known and potential confounders to the outcomes of bacterial sepsis would be assessed in all participants, and adjustment for confounding would be performed using logistic regression and/or stratification±Mantel-Haenszel estimator where applicable.

**Ethics and dissemination:**

Ethical approvals were granted by the University of Birmingham (ERN_2115-Jun2024), the Ahmadu Bello University Teaching Hospital (ABUTHZ/HREC/H45/2023), Barau Dikko Teaching Hospital, Kaduna (NHREC/30/11/21A) and the Kaduna State Ministry of Health (MOH/AD M/744/VOL.1/1110018). The study will be conducted using the international guidelines for good clinical practice and based on the principles of the Declaration of Helsinki. The results will be disseminated via oral and poster presentations in scientific conferences and published in peer-reviewed journal articles.

**STRENGTHS AND LIMITATIONS OF THIS STUDY**

- Data collection from four hospitals spread across four local government areas will ensure good generalisability of the findings in the state and applicability of the resulting recommendations.
- The cross-sectional design in this study ensures that clinical and laboratory data from each patient encounter are collected in detail in the case report forms, so that rare and unusual presentations could be published as case reports.
- In addition to determining the predictors of antibiotic-resistant genes, the cross-sectional design provides an opportunity for antibiotic-resistance surveillance during the study period.
- Patients will be recruited consecutively as they present, which could lead to selection bias, just as the caregivers’ responses on the questions of vaccination and socioeconomic status may be subject to recall and social desirability biases.
- This study design, being cross-sectional with no previous or baseline data for comparison, could be affected by confounders that are out of the scope of this project and therefore impossible to control.

## Introduction

### Paediatric sepsis

 Paediatric sepsis is defined according to the 2005 International Paediatric Sepsis Definition Consensus as having a systemic inflammatory response in the presence of a suspected or proven infection.^1^ Systemic inflammatory response syndrome is defined as a patient having at least two of the following criteria with one being either abnormal temperature or atypical white cell count. The criteria are fever, subnormal temperature or hypothermia; age-dependent tachycardia or bradycardia; tachypnoea; the need for mechanical ventilation; abnormal white blood cell count and >10% immature neutrophils.^1^ However, in low-income and middle-income countries (LMICs), the use of complete blood count for the initial diagnosis of sepsis is particularly challenging. This is so, because the facilities for a complete blood count may not be readily available, and where available, it usually takes at least 24 hours to obtain the result, leading to a delay in diagnosis. Hence, to facilitate quick diagnosis, this criterion is replaced by prostration.^2^ The major risk factors of paediatric sepsis include age, with the under-5s being more predisposed. Other predisposing factors include prematurity, low birth weight and socioeconomic status.^3^

### Burden of paediatric sepsis

Sepsis is of tremendous global health importance and is a leading cause of death and disability.^4^ Paediatric sepsis is responsible for an estimated 20 million annual deaths occurring in children globally, which translates roughly to a child dying of sepsis every 3 min.^5^ Apart from being a direct cause of morbidity and mortality, bacterial sepsis is also a common denominator in deaths associated with malignancies and other immunosuppressive illnesses.^6^ The prevalence and burden of sepsis varies considerably across age groups and world regions, with up to 85% of sepsis deaths taking place in LMICs, and children in sub-Saharan Africa being the most affected.^4^ Despite the enormity of these figures, sepsis burden is likely underestimated due to the paucity of data from sub-Saharan Africa and South-east Asia,^7^ and because the criteria for sepsis vary between countries and locations.^6^ Additionally, the treatment of bacterial sepsis has become complicated by the rising incidence of antimicrobial resistance (AMR). In 2019, estimates at a global scale indicated that up to 1.27 million sepsis deaths were attributable directly to AMR, which is projected to increase if unchecked.^8^

### Sepsis and antibiotic resistance in Nigeria

Nigeria is the second-highest contributor to global sepsis deaths, most of which are from the paediatric age group.^3^ Accordingly, the country recorded an estimated 5 333 767 (Uncertainty Interval: 3 474 576–8 085 457) cases of sepsis in 2017 alone.^5^ Despite this, paediatric sepsis is understudied in the country and the few available studies are mainly on neonatal sepsis.^9^ Again, like in other resource-constrained countries, it is challenging to establish the true prevalence of paediatric sepsis. Consequently, it was estimated that neonatal sepsis was being under-reported by at least 20%.^10^ Nonetheless, an analysis of the records of 54 secondary and tertiary hospitals across Nigeria between September 2019 and 31 August 2020 found a neonatal sepsis prevalence of 16.3 per 1000 live births with a 10.3% case fatality rate.^11^

More locally, a study of community-acquired bloodstream infections in Zamfara, northwest Nigeria, demonstrated a prevalence of paediatric sepsis of 20%.^12^ Further, a study at the Ahmadu Bello University Teaching Hospital (ABUTH), Zaria, northwest Nigeria in 2008 found an incidence of neonatal sepsis of 17.6 per 1000 live births.^13^ A similar study conducted in the same centre 10 years later found an incidence of neonatal sepsis of 27.3%.^14^ Moreover, the latest review of admissions into the neonatal unit of the same hospital in 2020 revealed an incidence of 37.6%.^15^ This trend suggests that the incidence of neonatal sepsis is increasing in Kaduna State, and given that the reporting system has not changed, the increased prevalence may be due to recent escalation in antibiotic resistance.^12^

The impact of antibiotic resistance is disproportionately felt in developing countries. Here, the problem of AMR is happening on the background of, and it is potentiated by poverty, malnutrition and limited availability and economic access to second-line antibiotics.^16^ Thus, in West Africa, in 2019, antibiotic-resistant sepsis alone was responsible for 412 000–663 000 deaths.^16^

In Nigerian communities, antibiotic misuse is very common as they are sold over the counter with or without a prescription.^17^ Also, less than a quarter of Nigerian tertiary healthcare facilities have antibiotic stewardship programmes, most of which are largely based on anecdotal evidence.^18^ There is also a paucity of reliable data concerning the magnitude, as well as the implications of antibiotic resistance in Nigeria.^19^ Furthermore, in resource-constrained settings, the situation is complicated by a lack of standardised facilities for the molecular diagnosis of antibiotic resistance.^20^

### Rationale for the study

There has been an increase in the prevalence of sepsis caused by antibiotic-resistant bacteria in sub-Saharan Africa, including Zaria, and the prevalence is expected to rise.^16^ The impact of AMR on the clinical outcome of children with sepsis has never been documented in Kaduna State. Further, the genomic epidemiology of bacteria causing sepsis in children in Nigeria and Kaduna State in particular is largely unknown. This study aims to determine the prevalence of culture-proven and antibiotic-resistant sepsis in children admitted with suspected sepsis in four high-patient-turnover hospitals in Kaduna State. It will also investigate the clinical characteristics of paediatric sepsis, the phenotypic features of the isolated bacteria and their antibiotic resistance patterns. It will further provide clinically relevant data as to the important determinants of sepsis from integrating clinical, haematological, as well as the phenotypic characteristics and genomic composition of the bacterial isolates. It will likewise provide evidence for effective antibiotic stewardship programmes in Kaduna State and by extension Nigeria.

## Materials and methods

### Study area

The study is being conducted at the ABUTH, Barau Dikko Teaching Hospitals (BDTH), Yusuf Dantsoho Memorial Hospital (YDMH) and Gambo Sawaba General Hospitals, all in Kaduna State, Nigeria. Kaduna is the third most populated state in Nigeria with a population of over 10 million inhabitants, with up to 60% being children under the age of 24 years and the largest proportion (20%) being the under-5s.^21^ The ABUTH and Gambo Sawaba General Hospitals are in Zaria, which is the second largest metropolis after the state capital, with a population of ~8 00 000. BDTH and YDMH Hospitals are situated in the Kaduna metropolis which has a total population of 1.85 million.^21^ ABUTH and BDTH provide secondary and tertiary healthcare services to residents in Kaduna and Zaria and serve as reference hospitals for the entire Kaduna state and the northwest region of the country. ABUTH has a total bed capacity of 650 and its paediatric department has a bed capacity of 100 and admits up to 2500 patients annually.^22^ BDTH has a total bed capacity of 240 and paediatric and neonatology wards admit up to 1200 patients annually.^23^ Both Gambo Sawaba and YDMHs have fewer bed capacities and admit up to 800 children annually. (Anecdotal) Up to 15.7% of Kaduna township residents who access care in these hospitals live in slums with poor environmental, water, personal and food hygiene.^24^ Laws regulating antibiotic use are not routinely enforced in the communities which fuels misuse.^17^

### Study population

The study will recruit any child aged below 18 years presenting with clinical features suggestive of sepsis. Parents or caregivers in this setting are of diverse ethnicities, but all of them either speak the local lingua franca (Hausa) or English.

### Study design and sample size

A cross-sectional hospital-based study design will be employed.

The sample size will be determined by the formula^25^:


$$n_{0}=\frac{Z^{2}pq}{d^{2}}$$

Where n_0_=minimum sample size

Z=normal deviate corresponding to 95% CI, that is, 1.96

p=prevalence of antibiotic-resistant pathogens in children with sepsis: p=50% (assuming an unknown prevalence of antibiotic-resistant genes in patients to be recruited).

q=1 p, d=df=0.5, q=1–0.5=0.5 d=0.05 (5% precision)


$$n_{0}=\frac{(1.96{)}^{2}\times 0.5\times 0.5}{(0.05{)}^{2}}$$

$$n_{0}=384$$

Allowing for a 20% non-response rate (in the case of accidental spillage of sample, withdrawal of consent, etc).


$$n_{0}=384+76.8$$

$$n_{0}=461$$

### Inclusion criteria

1. Children presenting with fever (≥38°C) or subnormal temperature (≤36°C).
2. Body weakness.
3. Any other systemic symptom such as cough, diarrhoea, convulsion, etc.

### Exclusion criteria

1. Participants who have commenced antibiotics for >24 hours after presentation.
2. Neonates of mothers who received antibiotics within 24 hours prior to delivery.

### Subject recruitment

**Figure 1:**
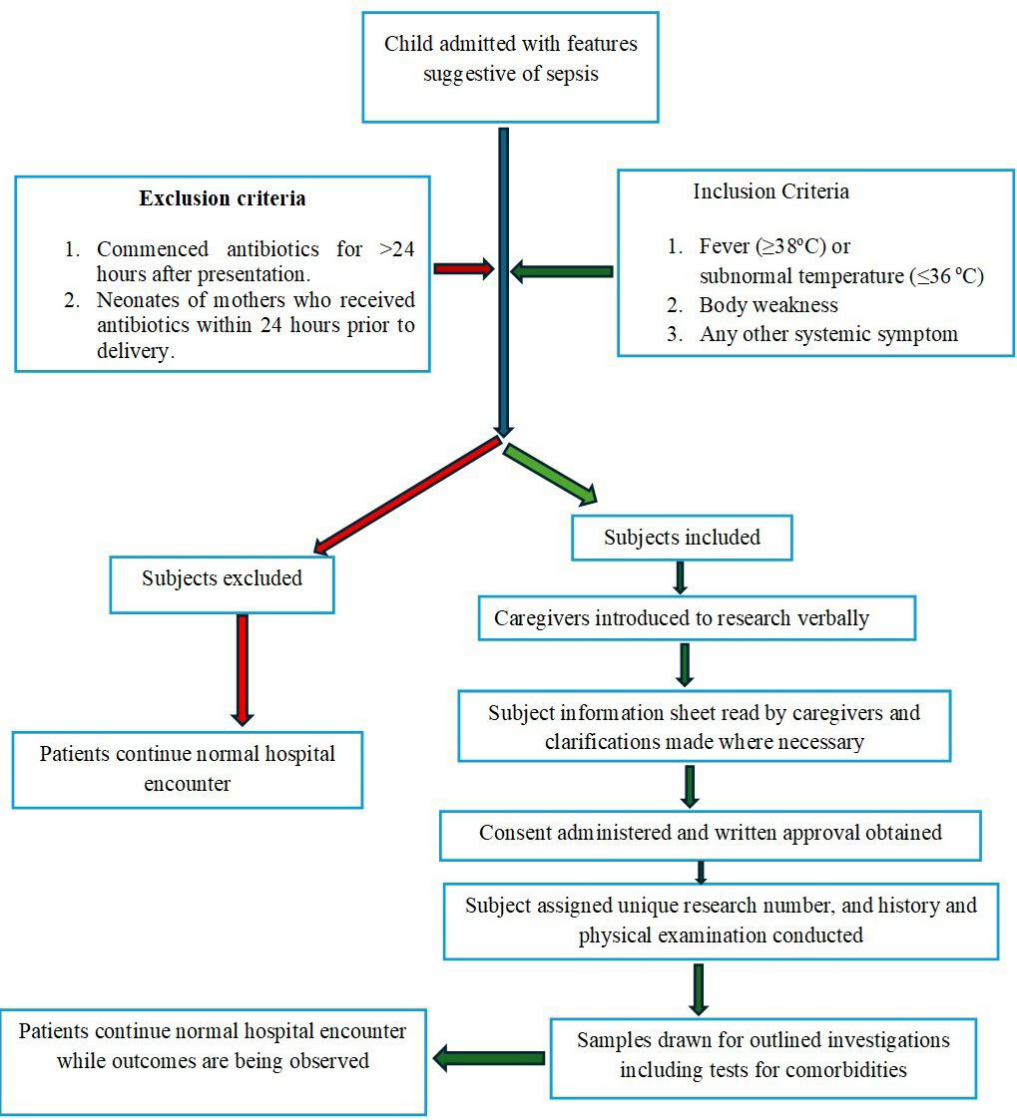
Schematic diagram of subjects’ recruitment.

The recruitment will be carried out consecutively at the different emergency points of entry into the hospitals until the desired sample size is attained. The notice of the research will be placed at the strategic places in the special care baby unit, emergency paediatric unit and surgical accident and emergency. The researcher will be notified directly or by phone once a child with suspected sepsis is to be admitted. The researcher or his assistants will then assess each patient against the inclusion and exclusion criteria to confirm suitability for inclusion or otherwise. Once a patient satisfies the inclusion criteria, the researcher will introduce the project to the caregivers verbally and subsequently give them the subject information sheet (online supplemental Appendix-ia) or its Hausa translation (online supplemental Appendix-ib) depending on which one the parent/caregiver understands better. The researchers will then ask questions to confirm that the caregivers understand the subject information sheet and make clarifications where necessary. Consent forms (online supplemental Appendix-ii) will then be administered to the caregivers and written endorsement obtained. In addition, assent will be gotten from children 7 years and older, after which the assent forms (online supplemental Appendix-iii)will be administered for endorsement. Thereafter, a unique research number is given to the subject, and history and examination will be carried out and the findings recorded in the proforma. Additionally, nutritional and haemoglobin SS status, known congenital immunosuppressive diseases, HIV infection and malaria would be determined and documented. The subject recruitment is presented in figure 1.

### Data collection

A structured, interviewer-administered questionnaire (online supplemental Appendix-iv) will be used to collect information on the child’s biodata, presenting symptoms and signs, preadmission antibiotics, vaccination status and parent’s socioeconomic status. Results of the complete blood count and bacterial isolates, antibiotic susceptibility test results and the records will be linked to the results of bacterial genetic analyses.

The proforma will then be transcribed into an electronic version and stored as a Word document by the researcher. All data will be entered into an SPSS spreadsheet (Statistical Package for Social Sciences (SPSS) V.27 (IBM)), as the data collection progresses, and access will be restricted to only the research team members. The data collection is expected to last for a period of 8 months from December 2024 to July 2025.

### Determination of socioeconomic status

Socioeconomic status will be determined using the method described by Ogunlesi *et al*^26^ which uses the educational status and occupation of the parents/caregivers to categorise children into five socioeconomic classes as shown in table 1.

**Table 1:** Socioeconomic scoring of educational qualification and occupation

Educational qualification of parents/caregivers	Occupation of parents/caregivers	Scores
PhD, Masters	Professionals and HST	1
Bachelor, higher national diploma	SGE (senior government employee)	2
OND, NCE, technical education	Clergy, HSF, JGE, MST, teachers, technicians, retirees	3
SSCE, JSCE*, grade II teaching certificate	Artisans, sentries and security agents	4
Primary, no formal education	Labourers, messengers, apprentices, students, peasant farmers and the unemployed	5

* Junior Secondary Education.

HSF, high-scale farming; HST, high-scale trading; JGE, junior government employee; JSCE, junior secondary education; MST, middle-scale trading; NCE, national certificate of education; OND, ordinary national diploma; SGE, senior government employee; SSCE, senior secondary education.

### Anthropometric measurement

The anthropometric parameters will be measured using the methods described by Cogill^27^ and all measurements will be performed twice to get the average. Values of weight and length for age less than −3 SD from the mean for age and sex of a child using the WHO Anthro Software V.3.2.2, 2010^28^ will be regarded as severe under-nutrition. Values less than −2 but not greater than −3 SD will be regarded as moderate undernutrition. While values greater than or equal to −2 and less than or equal to +2 SD will be considered normal. Values greater than +2 SD will be regarded as overweight/obesity.

### Specimen collection and processing

All the recruited patients will have blood samples collected into the BD BACTEC Peds Plus culture medium (Becton Dickinson & Co., USA, 20124) (online supplemental Appendix-v). The samples will be taken within the first 24 hours of admission and before the commencement of antibiotics. A 2 mL of blood will be inoculated into the blood culture bottle with the remainder used for complete blood count and blood film analysis.

### Processing of blood culture samples

Within 1 hour of collection, all blood culture samples will be transported to the medical microbiology laboratory and incubated at 37℃ in a BACTEC machine (Becton Dickinson BACTEC 9050 Blood Culture System). The BACTEC machine will indicate a positive sample with a red light at the front part of the cover and a negative sample by a green light. The green light is indicated after 5 days’ incubation period, and the negative sample is then destroyed. If any growth is observed, a Gram stain will be performed. The specimen will then be subcultured in MacConkey, Blood or Mueller Hinton agars and incubated aerobically at 37°C for 18–24 hours. Bacterial isolates will be stored in duplicate in Tryptic Soya Broth fortified with 20% glycerol at –80°C. Further identification of the bacteria will be conducted using identification kits including MICROBACT 12AX2, MICROBACT 12BX1 and MICROBACT Staph 12SX1.

### Antibiotic susceptibility tests

Antibiotic sensitivity testing will be conducted using the Kirby-Bauer method utilising the Oxoid antibiotic susceptibility test kit (Basingstoke, UK) and according to the Clinical and Laboratory Standards Institute.^29^ Antibiotics to be tested will include ampicillin 25 µg, cloxacillin 5 µg, chloramphenicol 10 µg, ceftriaxone 30 µg, ciprofloxacin 5 µg, gentamicin 10 µg, erythromycin 10 µg, penicillin, meropenem, colistin, linezolid, tazobactam/piperacillin, vancomycin and doxycycline. *Staphylococcus aureus* and *Escherichia coli* will be used as controls for Gram-positive and Gram-negative organisms respectively. For each tested antibiotic, isolates will be classified as susceptible, indeterminate or resistant based on the diameter of the disc.

### Complete blood count, blood film and HIV test

Blood sample for complete blood count will be analysed using a Norma Icon-3 automated 5-part differential haematology analyser (Norma Instruments Zrt, Hungary). The analyser gives absolute values as well as the differential percentages for neutrophils, eosinophils, basophils, monocytes and lymphocytes. This is in addition to the red blood cell indices and the platelets count.

Two blood films will be prepared from each sample using the standard wedge technique as described by Briggs *et al.*^30^ The first film will be stained with Leishman stain for 2 min and used to examine the morphology of the red and white blood cells as well as reticulocyte count. The second film will be flooded with methanol, dried and then stained with Giemsa for 20 min and washed in a water buffer, dried and examined for malaria parasites. Reading of the slides will be assisted by a consultant haematologist using a multiviewer light microscope (Leica Microsystems Wetzlar, Germany). The number of nucleated red cells per 100 leucocytes, differential neutrophil count and immature neutrophil count will be counted manually (with the aid of a manual counter).

A rapid serial testing algorithm will be used to screen the participants for HIV according to the Nigerian National HIV Testing Algorithm.^31^ Accordingly, each participant would first be screened using the Determine kit (Determine HIV-1/2 Ag/Ab Combo) and reported if positive. If negative, however, the test would be repeated using the Uni-Gold kit (Uni-Gold HIV) and the Stat-Pak kit (HIV 1/2 STAT-PAK Assay), where applicable, and all tests would be carried out according to the manufacturer’s guidelines.

### Genome sequencing for antibiotic resistance genes

Selected bacterial isolates will be sequenced using a third-party commercial provider to generate paired-end Illumina data according to manufacturer’s protocols. Genomic data will be used to confirm species/strain identity and screen for AMR markers.

### Dissemination of results

The Strengthening the Reporting of observational studies in Epidemiology (STROBE) initiative: STROBE guidance will be used to report the study findings.^32^ Results of this study will be presented at local and international seminars, workshops and conferences as well as articles in peer-reviewed, open-access journals.

### Data management and analysis

All data will be stored in link-anonymised format using paper case report forms stored securely at the research site. Fully anonymised sequencing data will be uploaded to a curated online data repository. Research findings will be published in peer-reviewed journals as soon as is practicable, with an online link to the final approved study protocol. Participants will be provided with a lay summary of the study results once available. The completed questionnaires will be checked for errors, and the information will be transferred into the statistical software for the SPSS V.27, cleaned and analysed.

The prevalence of culture-proven sepsis will be determined by dividing the number of patients with culture positive results by the total number of patients admitted and expressed in percentage. Subsequently, the prevalence of antibiotic-resistant sepsis will be established by analysing the antibiotic resistance pattern of each isolate. Likewise, measures such as antibiotic resistance spectrum and drug resistance index will be used to further characterise antibiotic resistance.

Data collated from the clinical, haematological, bacterial phenotypes and genomic variables will be expressed as text, numbers and proportions and presented in prose, figures, graphs and tables as appropriate. χ^2^ or Fischer’s exact test will be used for comparative analysis of categorical variables depending on the size of the expected frequency in a cell. Multivariate logistic regression analysis will be used to find the predictors of antibiotic-resistant genes. A p≤0.05 will be considered statistically significant.

**Figure 2:**
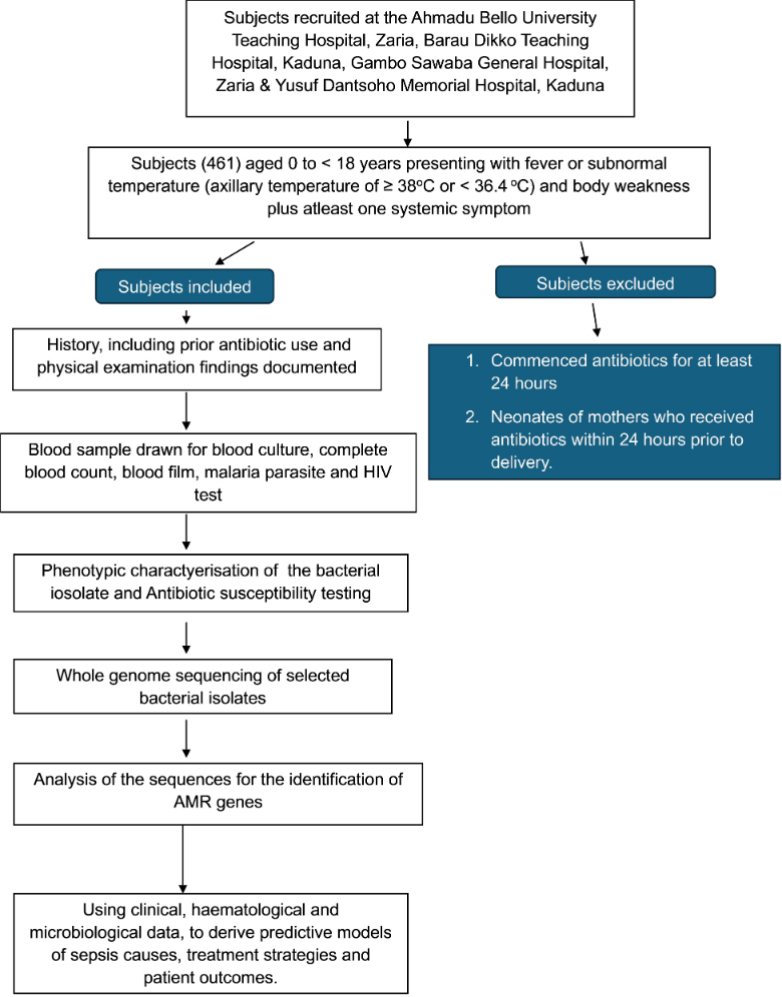
Flow chart of the methods. AMR, antimicrobial resistance.

Known and potential confounders to the outcomes of bacterial sepsis would be assessed in all subjects, and adjustment for confounding would be performed using logistic regression and/or stratification±Mantel-Haenszel estimator where applicable, as described by Pourhoseingholi *et al*.^33^ The methods are summarised in a flow chart in figure 2.

### Ethical issues and safety

Approval of the Research and Ethics Committees of the ABUTH (ABUTHZ/HREC/H45/2023) and BDTH (NHREC/30/11/21A), as well as the University of Birmingham (ERN_2115-Jun2024) has been obtained. Also, ethical approval for the use of the Gambo Sawaba General Hospital and YDMHs was granted by the Kaduna State Ministry of Health (MOH/AD M/744/VOL.1/1110018). Informed written consent will be obtained from each parent/caregiver before enrolment into the study, and they will be free to opt out of the study unconditionally without being affected in any way. Assent will also be obtained from all children 7 years or older. The research will not affect the standard of care for the subjects and will be conducted at no cost to subjects. The virtue of honesty and all the provisions of the Declaration of Helsinki will be duly observed.^34^

## Supplementary material

10.1136/bmjopen-2025-106612 online supplemental file 1
