# Supplementary material for: From bedside to bug side: clinical, haematological and genetic markers of antibiotic-resistant bacterial isolates from children admitted with sepsis in Kaduna State, Nigeria – a protocol for a cross-sectional study
Source: BMJ Open. 2025 Sep 26;15(9):e106612. doi: 10.1136/bmjopen-2025-106612 (PMC12481344; doi:10.1136/bmjopen-2025-106612)
Supplement: online supplemental file 1 [file bmjopen-15-9-s001.docx]

Appendix-ia

**Subject’s information sheet**

Thank you for agreeing to meet me today. My name is Sani Musa; I am a doctor in the Paediatric department of this hospital, and at the same time a PhD student with University of Birmingham. Just like you know, this is one of the centres in this country where children with serious infection are managed. To improve this service, we would want to improve our understanding of how the bugs interact with antibiotics. This will help us to better manage difficult-to-treat infections which often results in death or disability in the affected children. I am asking whether you will be able give consent for your child to participate in this study. As a participant in this study, relevant history will be obtained from you and your child (if older than 7 years and conscious enough). The child will also have physical examination conducted and the signs obtained documented. Further, the blood sample that has been withdrawn as part of your child’s routine care will be analysed for blood cells count and nature as well as isolate the bug that is causing the infection. These investigations are routinely carried out as part of ‘sepsis work up’ for all patients presenting with clinical features of infection. The bug will further be studied to understand how it interreacts with common antibiotics and also identify its component that are responsible for making it difficult to treat with antibiotics. Part of the test will be conducted here while a part will be conducted at the University of Birmingham in the United Kingdom. Information obtained from your child will remain anonymous as the identity of your child will not be made public. The information about your child that will be obtained will only be used for the purpose of this study, and no information will be traced back to your child except where your child is in immediate danger. In such situation, we will discuss with you and proffer the best available solution to your child. Your child’s participation in this study will not be at any financial cost to you. You are free to decline participation in this study on behalf of your child, and that is not going to affect the care that your child/ward will receive either now or in the future. If you have any question/concern about your child’s participation in this study, please contact:

Dr. Sani Musa, department of Paediatrics, ABUTH, Shika-Zaria

Phone no: 07039667354/+447438357996

Email: asmaummama@gmail.com/sxm2005@student.bham.ac.uk

Appendix-ib

Hausa translation of information sheet

Bayani akan wannan bincike ga iyaayen yara

Assalamu alaikum warahmatullahi wa barakatuh. Ina mika godiya ta gareku saboda amincewar ku muhadu a yau. Sunana Sani Musa. Ni likitane a bangaren yara na wannan asibitin. A halin yanzu, in yin digiri na uku a jamiar Birmingham da take kasar Biritaniya. Kamar yadda kuka sani, wannan asibitin yana cikin asibitocin dasuke kulawa da ciwuka masu tsanani dasuka shafi yara. A yunkurin inganta magance wadanan ciwuka, inaso in gudanar da bincike akan yanda kwayoyin cuta suke alaka da maganin da akansha. Fahimtar alakar kwayoyin cituttuka da magani zai karamana fahimtar yadda zamu magance irin wadannan ciwukan masu wahalan magancewa. Ciwukan da kan haddasa rasa rai ko nakasa. Ina tambayan izini na sanya yaronku ko yarinyarku cikin wannan binciken. Idan kun yarda, zanyi maku tambayoyi gameda rashin lafiyar sa/ta, sannan zan duba, da kuma aa auna jikinshi/ta. Sannan zan duba jinin sa/ta domin in duba yawanshi da kuma gano kwayoyin dasuka haddasa masa/ta ciwon. Shi dama irin wannan binciken akanyi shi ne yau da gobe ga duk yaron da aka kawo asibiti da lalurar kamuwa da kwayoyin cuta. Kuma, zan bincika alakar dake tsakanin kwayoyin cutar dakuma magungunar daakan sha domin su. Wannan zai taimakamun wajen fahimtar dalilin dayasa wadannan kwayoyin cutar sukan bada wahala wajen magancewa. Akwai bangaren wannan binciken da zanyi a wannan asibitin, sannan akwai wanda zanyi a jamiar Birmingham ta kasar Britaniya. Duk bayanan daka bayar gameda yaron/yarinyar ki zasu kasance a cikin sirri, kuma zaayi amfani dasu kadai saboda wannan binciken. Zan duba sirrin sannan na nemiku kadai idan anci karo da matsalar wanda ya kamata a baiwa yaron/yarinyar ku kulawa ta musamman. Bazan caje ku wani kudi ba saboda dan/diyar ku tashiga wannan binciken. Kuna iya kin yardarku ko yaronku/yarinyar ku su shiga wannan binciken, kuma kin yardar ku ba zai shafi yanda zamu kula da yaranku ba ayanzu, ko anan gaba. Idan kuna bukatan Karin bayani sai ku tuntubi;

Dr. Sani Musa, sashin kula da yara na asibitin koyarwa na Ahmadu bello (ABUTH) dake shika, (ABUTH) Zaria

Numbar waya: 07039667354/+447438357996

Email: asmaummama@gmail.com/sxm2005@student.bham.ac.uk

Appendix-ii

**Consent form**

***Clinical, haematological and genetic markers of antibiotic-resistant bacterial isolates from children admitted with sepsis in Kaduna State, Nigeria.***

Participant Identification Number for this Study: ………………………..

I confirm I have read and understood the information sheet dated......................... for the above study.

I have had the opportunity to consider the information, ask questions and have had these answered satisfactorily.

YES

NO

I understand that participation in this study is voluntary, and I am free to withdraw consent on my child’s behalf at any time, without giving a reason, without any penalties.

No

Yes

I understand that data collected during the study, may be looked at by individuals at the University of Birmingham, Ahmadu Bello University Teaching Hospital, Barau Dikko Teaching Hospital, Kaduna and the Kaduna State Ministry of Health.

No

Yes

I, hereby, declare that I have not been subjected to any form of coercion or financial inducement in giving this consent.

YES

NO

I agree to the data about my child collected in this study being stored for further use in the future.

Yes

No

I agree to take part in this study.

Yes

No

(NB: Signing this consent does not affect your right to decline to take part in any future study.) Name and signature/thumb print of Parents /authorised caregiver ……………

Name and Signature of person obtaining consent …………………

Name of witness Signature of Witness…………………………..

*Date………………………………………………………………*

Appendix-iii

**Assent Form**

**Project Title:** *Clinical, haematological and genetic markers of antibiotic-resistant bacterial isolates from children admitted with sepsis in Kaduna State, Nigeria.*

**Investigator:** Dr Sani Musa

We are doing a research study about the sickness that is caused by infection. A research study is a scientific way to learn more about people. In this research, we will be studying those small bugs called bacteria that cause sickness and are treated with drugs known as antibiotics. The study will help us to understand how the bugs interreact with antibiotics so as to be able to manage the infections better. As a participant in this study, we would want to know more about your sickness and examine your body. Also, the blood sample that has been withdrawn as part of your routine care will be analysed for blood cells and to isolate the bug that is causing the infection and making you sick

Everyone who takes part in this study will **NOT** benefit directly. A benefit means that something good happens to you. The possible benefits from this study might be that the findings of the study will help us to improve the treatment of infections in the future, so that patients will recover faster. When we are finished with this study, we will write a report about what was learned. This report will not include your name or that you were in the study. You can be in this study if you want to be. If you decide to stop after we begin, that’s okay too. Your parents know about the study too.

If you decide you want to be in this study, please sign your name.

I, _________________________________, want to be in this research study.

| _______________________________ | ______ |
| --- | --- |
| (Sign your name here) | (Date) |
| ___________________________________ | ______ |
| (Signature of parent/ legally accepted guardian) | (Date) |
| ___________________________________ | ______ |
| (Signature of Witness) | (Date) |

**Appendix-iv**

**Sepsis Study-Questionnaire**

Serial Number-SS- Hospital no…………………….

Date and time of recruitment……………………………………………………….

Ward……………… Phone number of parents/Caregivers……………

Section 1: Demographic characteristics of the respondent

- 1. Name………………………………
  2. Age (months)………………………
  3. Sex………………………………….

1.3 Ethnicity………………………

1.5 Family size……………………

1.6 Number of rooms…………….

1.7 Residential Address including state and LGA………………………………………………

**Section 2: Socioeconomic status of respondent (using the method described by Ogunlesi *et al*)**

**Average family monthly income:**

Father’s Age (years)…..

Score Education status Occupation

1 [ ] Phd,Masters [ ] Professional, HST

2 [ ] Bsc,HND [ ] SnrGovt Employee

3 [ ] OND,NCE,Technical [ ] JGE,Clergy,MST,Retiree

4 [ ] SSCE,JSCE,Grade II [ ] Artisan, Security Agents

5 [ ] Primary, No formal Educ [ ] Labourer,Messenger,Peasant, umemployed

Mother’s age:

Score : Education status Occupation

1 [ ] Phd,Masters [ ] Professional, HST

2 [ ] Bsc,HND [ ] SnrGovt Employee

3 [ ] OND,NCE,Technical [ ] JGE,Clergy,MST,Retiree

4 [ ] SSCE,JSCE,Grade II [ ] Artisan, Security Agents

5 [ ] Primary, No formal Educ [ ] Labourer,Messenger,Peasant

Calculated Child’s Socioeconomic score

$\frac{Fathers Total + Mothers Total}{4}$ = Child’s Score

Child’s Socioeconomic class

a. Class I [ ] b. Class II [ ] c. Class III [ ] d. Class IV [ ] e. Class V

KEY

Phd= Doctor of Philosophy

HST= High scale Trading

JGE= Junior Government Employee

MST=Middle Scale Trading

HND= Higher National Diploma

**Section 3: Patients History in detail**

OND= Ordinary National Diploma

NCE= National Certificate of Education

SSCE= Senior Secondary Certificate

JSCE= Junior Secondary Certificate

1. Presenting complaints and their duration………………………………………
2. Additional symptoms........................
3. History of prior antibiotic use, their nature & duration of use………
4. Other Interventions before admission……………………………..
5. Risk factors for infection..................................................................
6. Food hygiene…………………………………………………..
7. Water hygiene…………………………………………………
8. Environmental hygiene……………………………………….
9. Sewage disposal………………………………………………
10. Contact with a person with similar infection…………………
11. Past medical history……………………………………………….
12. Vaccination history............................................................................
13. Development history...........................................................................
14. Other Family and social history………………………...

**Section 4: Patients detailed examination**

1. General Physical Examination

| Sign | Present | Severity if present | Absent |
| --- | --- | --- | --- |
| Pyrexia |  |  |  |
| Pallor |  |  |  |
| Dehydration |  |  |  |
| Cyanosis |  |  |  |
| Lymphadenopathy |  |  |  |
| Jaundice |  |  |  |
| Oedema |  |  |  |
| Sclerema |  |  |  |
| Petechial rashes |  |  |  |
| Mottled skin |  |  |  |
| Capillary refill |  |  |  |
| Other general physical signs |  |  |  |
|  | | | |
|  | | | |
|  | | | |

1. Anthropometry

Weight…………………………………… (Kg)

Heights/Length……………………..…… (cm)

MUAC…….………………………….….. (cm)

1. Nutritional status………………….

| 1. Systemic Signs |  |  |  |
| --- | --- | --- | --- |
| **Cardiovascular:** PR/HR- BP- AB- |  |  |  |
| Position of apex………  HS-  **Respiratory:** RR- BS- SPO2:  Additional sounds: |  |  |  |
| **Digestive:** Oral rashes- Oropharynx-  Abdominal girth- Liver- Spleen-  Bowel sounds- Rectal exam- |  |  |  |
| **Genitourinary:** Suprapubic tenderness- Renal angle tenderness-  Urethral/vulval discharge……………. |  |  |  |
| **Central Nervous System:** Irritability- Pupils- Meningeal signs-  Fontanelles- Tone- power |  |  |  |
| **Musculoskeletal:** Skin rashes- Bone tenderness-  Joint swelling & tenderness- |  |  |  |
| **Haematological:** |  |  |  |
| **Endocrine:** |  |  |  |

5: Complete blood count results, blood film and retics. Attached print out.

6: Bacterial isolates and susceptibility profile (see attached);

Present: yes no

If present, type of isolate:

1.

2.

Antibiotic susceptibility profile (see attached results).

7. Comorbidities

1. Malaria test result…………
2. HIV test result…………………
3. HB electrophoresis pattern……
4. RBS at admission……………
5. Other chronic conditions………
6. Nutritional status………………

z-score for weight…………..

z-score for height………

7. Clinical diagnosis

8.Complications……………………………………………………………………………………………………………………………………………………………………………………………………………………………………………………………………………………………………………………

1. Focus/foci of infection/systems involved…………………………….

7: Antibiotics used during admission, their dosages and duration of administration.

……………………………………………………………………………………………………………………………………………….………………………

………………………………………………………….

…………………………………………………………..

8: **Outcome:** a. Discharged with antibiotics b. Discharged without antibiotics d. LAMA e. Discharged with sequalae f. Died

9: **Duration of admission in hours**………………………

10: **Estimated treatment cost in NGN**………..

**Appendix-v**


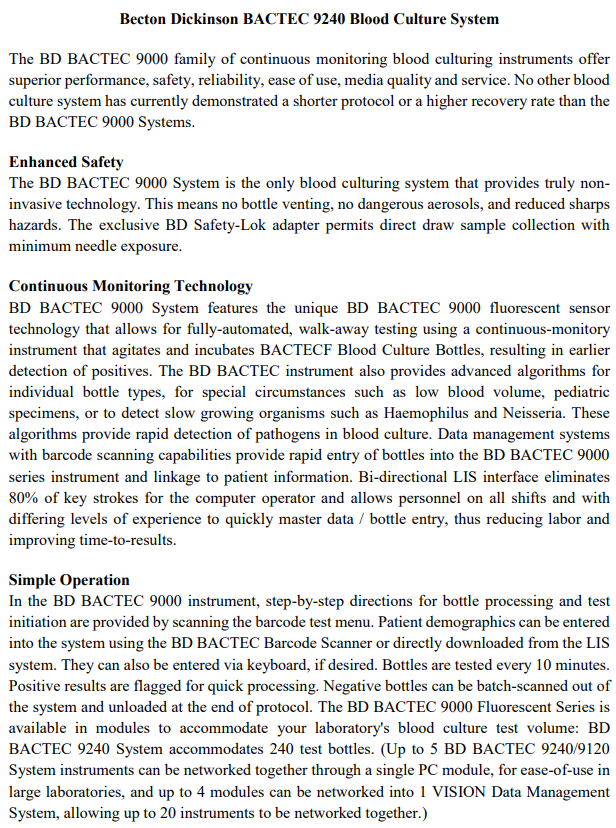

•
