# Supplementary material for: From bedside to bug side: clinical, haematological and genetic markers of antibiotic-resistant bacterial isolates from children admitted with sepsis in Kaduna State, Nigeria – a protocol for a cross-sectional study
Source: BMJ Open. 2025 Sep 26;15(9):e106612. doi: 10.1136/bmjopen-2025-106612 (PMC12481344; doi:10.1136/bmjopen-2025-106612)
Supplement: Supplementary file 2 [file bmjopen-2025-106612.reviewer_comments.pdf]

PEER REVIEW HISTORY

BMJ Open publishes all reviews undertaken for accepted manuscripts. Reviewers are asked to complete a checklist review form and are provided with free text boxes to elaborate on their assessment. These free text comments are reproduced below.

ARTICLE DETAILS

Title (Provisional)

FROM BEDSIDE TO BUG SIDE - CLINICAL, HAEMATOLOGICAL AND GENETIC MARKERS OF ANTIBIOTIC-RESISTANT BACTERIAL ISOLATES FROM CHILDREN ADMITTED WITH SEPSIS IN KADUNA STATE, NIGERIA: PROTOCOL FOR A CROSS-SECTIONAL STUDY.

Authors

Musa, Sani; Aliyu, Shamsudin; Abdullahi, Nuruddeen Bida; Khalid, Hajara Lawal; Salihu, Sadiq Kwaifa; Dahiru, Aliyu Umar; Muhammad, Amal Aminu; Abdullahi, Kamilah; Salisu, Suleiman; Gumbi, Summaiyah Ahmad; Tanko, Zainab Lamido; Umaru, Hauwau Makarfi; Bello-Manga, Halima; Dogara, Livingstone Gayus; Musa, Abdullahi; Usman, Ibrahim Kusfa; Lawal, Umar Waisu; Cleary, David W; Blair, Jessica

---

VERSION 1 - REVIEW

**Reviewer**	**1**
**Name**	**Timmesfeld, Nina**
**Affiliation**	**Ruhr-Universitat Bochum, Department of Medical Informatics, Biometry and Epidemiology**
**Date**	**08-Aug-2025**
**COI**	**None**

First of all, I would mentioned that my review focuses mainly on ensuring that all points relevant to a study protocol are reported in a clear manner. Since at the moment there are no special guidelines for study protocols observations studies, I will mainly consider theSTROBE guideline and some points from the SPIRIT guideline. Furthermore, there is actually a newly developed checklist for protocols of observation studies (SPIROS-Checklist) available under https://www.maastrichtuniversity.nl/file/spiros-checklist-v1pdf.

Here are my major comments:

1. First, in the study protocol (l. 227) it is stated that data collection will be done between December 2024 to July, 2025. From this statement, I would guess that recruitment will be finished by now. On the other hand, there are documents from the ethics committees from

the year 2025 which suggest that recruitment will be ongoing. This should be explained. Furthermore, I could not find any registration number so that I could validate the current trial status. Please state, if the trial is prospectively registered and in which register and give the registration number.

2. The study design (cross-sectional, cohort or case-control) should be given in the title and abstract. In l. 195 it is mentioned that this is a cross-sectional study. I am little unsure about this, because elsewhere it is reported that outcomes of the sepsis like death will be considered. This means that a follow-up of the patients will be done. In this case, this is a cohort study.

3. The main objective for the trial is unclear for me. From the sample size calculation, it seems that it is to estimate the prevalence of antibiotic-resistant pathogens in children with sepsis. But this is not mentioned on page 6, where according to the STROBE checklist the objectives are listed. Furthermore, the estimation of this prevalence is not mentioned in the statistical analysis section. Furthermore, the analysis provided there does not fit well with the objectives outlined under p. 6. Hence, the primary (main) and secondary objectives should clearly outlined at the end of the introduction (P.6) and the sample size calculation and statistical analysis section must match with these objectives.

4. The inclusion and exclusion criteria are not clearly described: “Children presenting with pyrexia and body weakness plus or minus tachycardia and/or bradycardia, tachypnoea, bradypnea or apnoea will be included into the study.” What is meant by plus or minus? Which condition has the children to fulfill? (1) tachycardia or bradycardia and (2) tachypnoea or bradypnea or apnoea? Both of them ((1) and (2))) or only one of them?

L. 214 “Subjects who fulfil the inclusion criteria will be given a unique identification number and recruited into the study.” Is this correct? At this time point no informed consent was available. L. 219 “The researcher or his assistants will then confirm the presence of sepsis or otherwise by conducting a thorough history and physical examination.” Is the presence of clinical confirmed sepsis an additional inclusion criterion which is applied in a second step? Please describe the recruitment and inclusion process more clearly and with more details. If it is a stepwise process, a flow chart might be helpful.

5. L. 485 is it correct, that the name is on the CRF?

Minor comments:

l. 57 perfumed

l.332 participants -> parents

l. 333 accent ->consent

VERSION 1 - AUTHOR RESPONSE

Reviewer: 1
Prof. Nina Timmesfeld, Ruhr-Universitat Bochum
Comments to the Author:
First of all, I would mention that my review focuses mainly on ensuring that all points relevant to a study protocol are reported in a clear manner. Since at the moment there are no special guidelines for study protocols observations studies, I will mainly consider the STROBE guideline and some points from the SPIRIT guideline. Furthermore, there is actually a newly developed checklist for protocols of observation studies (SPIROS-Checklist) available under https://www.maastrichtuniversity.nl/file/spiros-checklist-v1pdf.

Here are my major comments:
1. First, in the study protocol (l. 227) it is stated that data collection will be done between December 2024 to July 2025. From this statement, I would guess that recruitment will be finished by now. On the other hand, there are documents from the ethics committees from the year 2025 which suggest that recruitment will be ongoing. This should be explained.

We apologise for the lack of clarity here. The ethical approvals were not aligned with the data collection period. The ethics covered a period longer than the stated period for data collection. This is to cover for the extra-time that may be needed in case of unforeseen circumstance such as strike actions by health workers, low patients turn out, etc. Specifically, the ethics by the Ahmadu Bello University Teaching Hospital, Zaria was given for 2024 and had to be renewed for 2025.

Furthermore, I could not find any registration number so that I could validate the current trial status. Please state, if the trial is prospectively registered and in which register and give the registration number.

We thank the reviewer for this question. To clarify, this study is not a clinical trial and as such it was not registered. There are no controls and there was no exposure(s) of interest or intervention(s) of any sort administered. The protocol is being published to prevent duplication and future publication bias.

2. The study design (cross-sectional, cohort or case-control) should be given in the title and abstract.

The study design which is cross-sectional is now mentioned in the abstract and added to the title (see title & page 2: l.43)

In l. 195 it is mentioned that this is a cross-sectional study. I am little unsure about this, because elsewhere it is reported that outcomes of the sepsis like death will be considered. This means that a follow-up of the patients will be done. In this case, this is a cohort study.

The outcomes that were being referred to are the outcomes of a single hospital admission which could be normal discharge, discharge against medical advice or death. The study focussed on only patients who were admitted with sepsis and all the data collected on the patients relates to the single hospital encounter during which the patient is admitted. There

was no follow-up at any time after discharge. Also, unlike cohort study, there were no controls, no defined exposures or interventions and no specific outcome of interest. We hope this addresses this question.

3. The main objective for the trial is unclear for me. From the sample size calculation, it seems that it is to estimate the prevalence of antibiotic-resistant pathogens in children with sepsis. But this is not mentioned on page 6, where according to the STROBE checklist the objectives are listed.

Yes, the reviewer is correct that the main objective of the study is to 'to determine the prevalence of culture proven and antibiotic-resistant sepsis in children admitted with suspected sepsis in four high-patient-turnover hospitals in Kaduna State.' To reinforce this narrative, we have added further text on page 7. l.168-171.

Furthermore, the estimation of this prevalence is not mentioned in the statistical analysis section.

We apologise for this omission. This has now been added as quoted below.

'The prevalence of culture proven sepsis will be determined by dividing the number of patients with culture positive result by the total number of patients admitted and expressed in percentage. Subsequently, the prevalence of antibiotic-resistant sepsis will be established by analysing the antibiotic resistance pattern of each isolate. Likewise, measures such as antibiotic resistance spectrum and drug resistance index will be used to further characterise antibiotic resistance.'

See page 14: l.377-383

Furthermore, the analysis provided there does not fit well with the objectives outlined under p. 6. Hence, the primary (main) and secondary objectives should clearly be outlined at the end of the introduction (P.6) and the sample size calculation and statistical analysis section must match with these objectives.

This has now been corrected and clarified in the text, See page 7, l.168-171 & page 14, l.377-383

4. The inclusion and exclusion criteria are not clearly described: "Children presenting with pyrexia and body weakness plus or minus tachycardia and/or bradycardia, tachypnoea, bradypnea or apnoea will be included into the study." What is meant by plus or minus? Which condition has the children to fulfill? (1) tachycardia or bradycardia and (2) tachypnoea or bradypnea or apnoea? Both of them ((1) and (2))) or only one of them?
L. 214 "Subjects who fulfil the inclusion criteria will be given a unique identification number and recruited into the study." Is this correct? At this time point no informed consent was

available. L. 219 “The researcher or his assistants will then confirm the presence of sepsis or otherwise by conducting a thorough history and physical examination.” Is the presence of clinical confirmed sepsis an additional inclusion criterion which is applied in a second step? Please describe the recruitment and inclusion process more clearly and with more details. If it is a stepwise process, a flow chart might be helpful.

We thank the reviewer for this suggestion. These have now been clarified in the text, and a schematic diagram has been included as suggested and uploaded as separate files in the submission system.

5. L. 485 is it correct, that the name is on the CRF?

Yes, the subject name, hospital number and the unique research numbers were on the case report forms initially, this was to ease monitoring of the patient for admission outcomes and to link the presentation, laboratory and outcome data. Subsequently, the data was converted to electronic form and anonymised by removing the names and hospital numbers.

Minor comments:
l. 57 perfumed

Spelling corrected to 'performed' see page 2: (l.47)

l.332 participants -> parents

This has been corrected to parents/caregivers see page 16: l.404

l. 333 accent ->consent

This has been corrected to 'assent' which is applicable to children above seven years and is seeking the willingness of the children to partake in the research even though they cannot legally consent. see page 16: l.405
Reviewer: 1
If you have selected 'Yes' above, please provide details of any competing interests.: No competing interests
